# Disagreement in cardiac output measurements between fourth-generation FloTrac and critical care ultrasonography in patients with circulatory shock: a prospective observational study

**DOI:** 10.1186/s40560-019-0373-5

**Published:** 2019-04-11

**Authors:** Thomas Kaufmann, Ramon P. Clement, Bart Hiemstra, Jaap Jan Vos, Thomas W. L. Scheeren, Frederik Keus, Iwan C. C. van der Horst, SICS Study Group, Geert Koster, Frederik Keus, Iwan C. C. van der Horst, Willem Dieperink, Roos Bleijendaal, Yasmin F. Cawale, Ramon P. Clement, Devon Dijkhuizen, Ruben J. Eck, Bart Hiemstra, Anja Haker, Casper D. H. Hilbink, Thomas Kaufmann, Martiene Klasen, Manon Klaver, Laura J. Schokking, Victor W. Sikkens, Madelon Vos, Justin Woerlee, Renske Wiersema

**Affiliations:** 1 Department of Anesthesiology, University Medical Center Groningen, University of Groningen, P.O. Box 30.001, 9700 RB Groningen, The Netherlands; 2 Department of Critical Care, University Medical Center Groningen, University of Groningen, Groningen, The Netherlands

**Keywords:** Cardiac output, Critical care ultrasonography, Intensive care, Critically ill, Shock, Monitoring, Pulse contour analysis

## Abstract

**Background:**

Cardiac output measurements may inform diagnosis and provide guidance of therapeutic interventions in patients with hemodynamic instability. The FloTrac™ algorithm uses uncalibrated arterial pressure waveform analysis to estimate cardiac output. Recently, a new version of the algorithm has been developed. The aim was to assess the agreement between FloTrac™ and routinely performed cardiac output measurements obtained by critical care ultrasonography in patients with circulatory shock.

**Methods:**

A prospective observational study was performed in a tertiary hospital from June 2016 to January 2017. Adult critically ill patients with circulatory shock were eligible for inclusion. Cardiac output was measured simultaneously using FloTrac™ with a fourth-generation algorithm (CO_AP_) and critical care ultrasonography (CO_CCUS_). The strength of linear correlation of both methods was determined by the Pearson coefficient. Bland-Altman plot and four-quadrant plot were used to track agreement and trending ability.

**Result:**

Eighty-nine paired cardiac output measurements were performed in 17 patients during their first 24 h of admittance. CO_AP_ and CO_CCUS_ had strong positive linear correlation (*r*^2^ = 0.60, *p* < 0.001). Bias of CO_AP_ and CO_CCUS_ was 0.2 L min^−1^ (95% CI − 0.2 to 0.6) with limits of agreement of − 3.6 L min^−1^ (95% CI − 4.3 to − 2.9) to 4.0 L min^−1^ (95% CI 3.3 to 4.7). The percentage error was 65.6% (95% CI 53.2 to 77.3). Concordance rate was 64.4%.

**Conclusions:**

In critically ill patients with circulatory shock, there was disagreement and clinically unacceptable trending ability between values of cardiac output obtained by uncalibrated arterial pressure waveform analysis and critical care ultrasonography.

**Trial registration:**

Clinicaltrials.gov, NCT02912624, registered on September 23, 2016

**Electronic supplementary material:**

The online version of this article (10.1186/s40560-019-0373-5) contains supplementary material, which is available to authorized users.

## Background

Critically ill patients with circulatory shock have increased risks of multi-organ failure, long-term morbidity, and mortality [1]. Advanced hemodynamic monitoring in these patients may inform diagnosis and simultaneously guide management by providing insight into cardiac function, cardiac preload, and afterload [2]. Several methods for measuring cardiac output (CO) exist, varying from invasive (e.g. thermodilution by pulmonary artery catheter (PAC)) to minimally invasive (e.g. pulse contour analysis by FloTrac™ (Edwards Lifesciences, Irvine, USA)) or even non-invasive (e.g. transthoracic Doppler ultrasound by critical care ultrasonography (CCUS)). These methods all have their own merits, disadvantages and requirements [3].

One type of pulse contour analysis is the uncalibrated arterial pressure waveform analysis method to estimate CO (APCO). Reliability of APCO is questioned in patients with hemodynamic instability, and this occurs frequently in patients admitted to the ICU [4]. Therefore, CO measurements obtained by APCO should be interpreted with caution in critically ill patients with circulatory shock [5, 6].

The FloTrac™ system using the APCO method calculates CO based on the principle that aortic pulse pressure is proportional to stroke volume (SV) and inversely related to aortic compliance using a proprietary algorithm. FloTrac™ has been widely studied in more than 70 validation studies as of yet, mostly showing adequate performance in normo- and hypodynamic conditions, but not in patients with large changes in vascular tone which typically occur in patients with circulatory shock [7]. However, these studies vary by the statistical methods and versions of the algorithm used. Recently, the fourth-generation algorithm was developed to improve performance.

Evaluation of the trending ability rather than the agreement of absolute values of CO monitoring devices is increasingly considered in validation studies for assessment of potential clinical usefulness [8]. In addition to one single CO measurement for diagnosing circulatory shock, repeated measurements of CO informing the trending ability could be informative for monitoring and guidance of supportive treatments of patients with circulatory shock.

The aim of our study was to compare both agreements and trending ability for APCO measurements of CO (CO_AP_) with CO routinely measured by CCUS (CO_CCUS_) in critically ill patients with circulatory shock. CCUS was chosen as the reference standard since it is the preferred method for diagnosis, but not for monitoring, of circulatory shock in critically ill patients and is widely available [2, 9]. Importantly, it should be noted that CCUS is not a gold standard reference technique for method comparison studies aiming to evaluate the validity of CO monitors [10].

## Methods

This study was a substudy of the Simple Intensive Care Studies-I (SICS-I), which was a single-centre, prospective, observational cohort study in which all consecutive acutely admitted adult patients expected to stay beyond 24 h were included (NCT02912624) [16, 17]. The STROBE guidelines for reporting observational studies were used (Additional file 1) [11]. The checklist for CO monitor method comparison studies was used [10]. The local institutional review board (Medisch Ethische Toetsingscommissie, University Medical Center Groningen) approved the study (M15.168207 and M16.193856). Written informed consent was obtained from all patients.

### Selection criteria

In this substudy, all consecutive acutely admitted adult patients with suspected circulatory shock and expected to stay beyond 48 h were included from June 2016 to January 2017. The circulatory shock was defined as the requirement of any dose of vasopressor to maintain a mean arterial pressure (MAP) of 60 mmHg or if the MAP remained below 70 mmHg despite fluid resuscitation (defined by at least 1000 mL of crystalloids). In addition, at least one other sign of organ or tissue hypoperfusion had to be present: altered state of mind (Alert-Voice-Pain-Unresponsive scale) [12], mottled skin (Mottling score ≥ 1 [13]), decreased urine output (≤ 0.3 mL kg^−1^ h^−1^) or increased serum lactate level (≥ 2 mmol L^−1^). Exclusion criteria were inability to obtain sufficient quality CCUS images; no arterial line; atrial fibrillation; and aortic valve or mitral valve diseases known to impair the arterial waveform. We included this group of patients because CO measurements are indicated to identify the type of shock, select necessary therapeutic interventions and evaluate patient’s response to therapy [2].

### Objectives

The primary objective was to evaluate CO_AP_ measurements in terms of the agreement and trending ability against CO_CCUS_ as reference technique in patients with circulatory shock.

### Definitions and bias

Patient characteristics including clinical, hemodynamic and laboratory variables as well as Acute Physiology And Chronic Health Evaluation (APACHE) IV and Simplified Acute Physiology Score (SAPS) II values were recorded [14, 15]. Measurements were performed following protocolized definitions and procedures [16, 17].

In short, CO_CCUS_ was measured by transthoracic echocardiography using the Vivid-S6 system (General Electric, Horton, Norway) with cardiac probe M3S or M4S, and with default cardiac imaging setting. The parasternal long axis was used to measure the left ventricular outflow tract diameter. In the apical five-chamber view, a pulse wave Doppler signal in the left ventricular outflow tract was used to measure the velocity time integral. CO_CCUS_ was calculated using an established formula [18]. CCUS was performed after ICU admission within 6 h and repeated once every 24 h after admission provided there was no interference with clinical care. Researchers were trained in performing CCUS by experienced cardiologist-intensivists.

The FloTrac™ sensor was connected to an indwelling radial artery catheter and an EV1000™ monitor (version 4.00; Edwards Lifesciences, Irvine, USA), which continuously displayed CO_AP_ values. The value of CO_AP_ displayed on the EV1000™ monitor was registered simultaneously (i.e. ‘beat-to-beat’) with each CO_CCUS_ measurement.

All measurements, including CCUS findings, were kept blind for the caregivers. Quality of CCUS images and CO_CCUS_ measurements were validated by an independent specialized core laboratory (Groningen Image Core Lab) blinded for the CO_AP_ measurements

### Statistical analysis

No formal sample size calculation was performed due to lack of data on CO_AP_ variation in patients with circulatory shock. Therefore, this study has an exploratory nature.

Data were presented as means with standard deviations or medians with interquartile ranges depending on distributions. Normality of data was checked using the Shapiro-Wilk test. Dichotomous and categorical data were presented in proportions.

Correlations were assessed by scatter plot, and the strength of linear correlation was determined by calculating a Pearson (*r*) coefficient. Bland-Altman analyses of repeated measurements in each patient were performed and means (bias) and SD of the differences, 95% limits of agreement (LOA) (=mean difference ± 1.96 × SD of the difference) as well as the percentage error of CO_AP_ versus CO_CCUS_ were calculated [19, 20]. To evaluate the trending ability of CO_AP_ versus CO_CCUS_ a four-quadrant plot was used and the concordance rate was calculated using an exclusion zone of 0.5 L min^−1^ [21]. For statistical analysis, we used STATA version 15.0 (StataCorp, College Station, USA).

## Results

### Participants

**Fig. 1:**
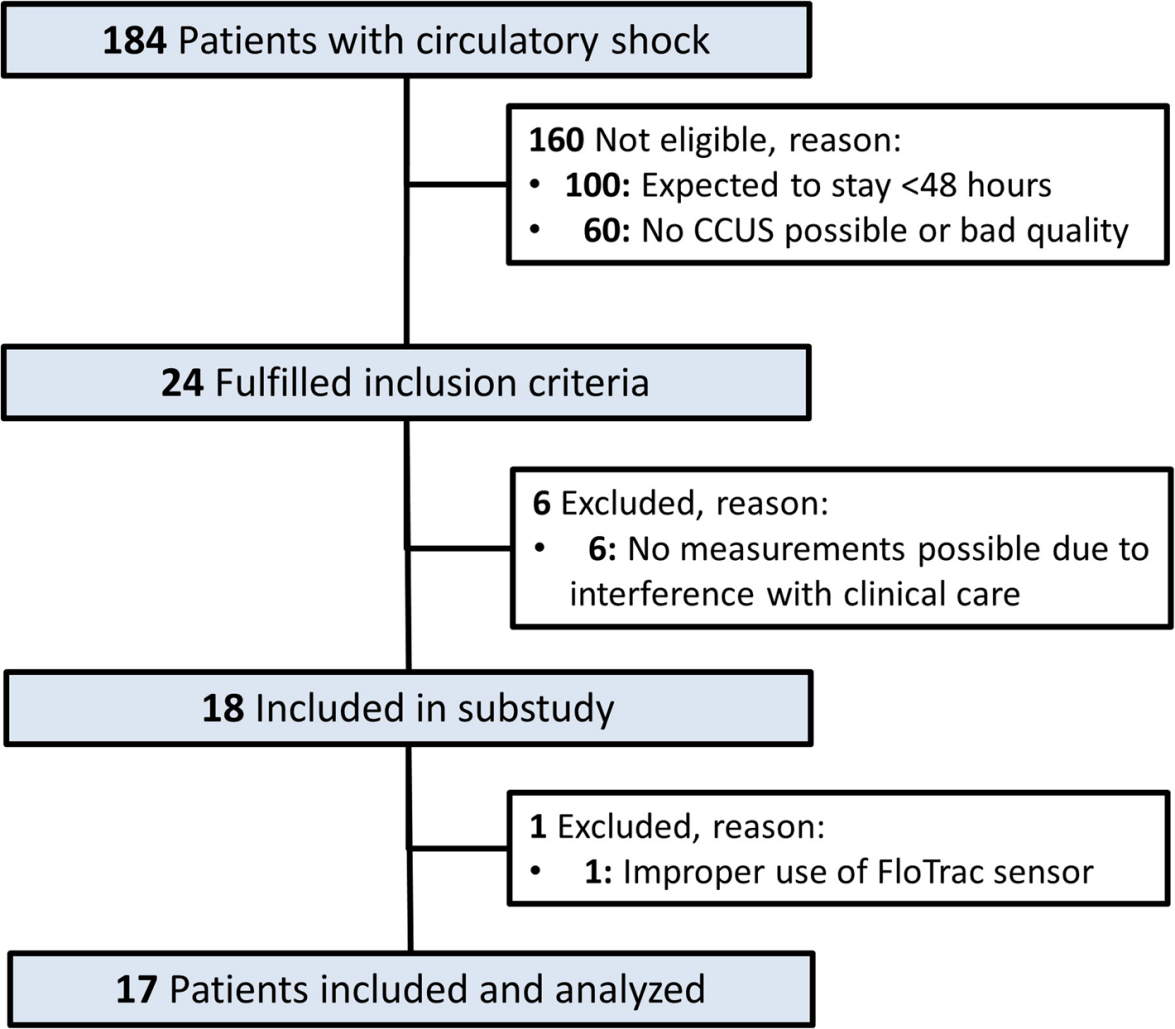
Patient flowchart. Abbreviations: CCUS, critical care ultrasonography

During the study period, 184 patients were diagnosed with circulatory shock, but only 24 patients appeared eligible for this study. One hundred patients who had circulatory shock were not included as they were expected to stay for less than 48 h, and 60 patients with circulatory shock were not included because CCUS was not possible or image quality was insufficient to perform measurements. Six patients had to be excluded because study procedures interfered with clinical care, leaving 18 patients to be included. One patient was excluded afterwards for invalid CO_AP_ measurements due to improper use of a FloTrac™ sensor. Thus, 17 patients were included in the final analyses (Fig. 1).

### Bias, precision and correlation

**Fig. 2:**
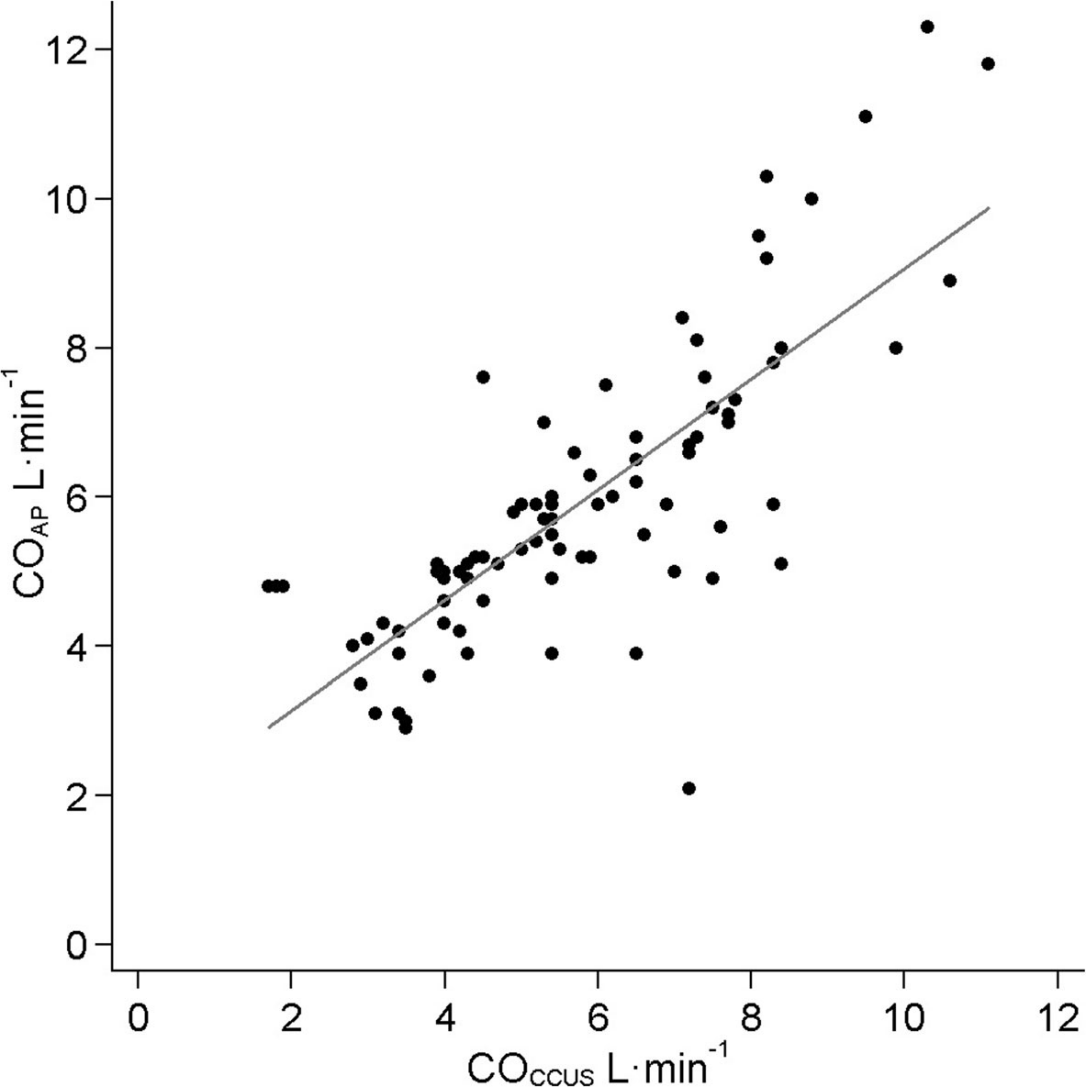
Scatter plot of cardiac output measured by FloTrac™ and CCUS. Abbreviations: CO_AP_, cardiac output measured using fourth-generation FloTrac™ algorithm; CO_CCUS_, cardiac output measured by critical care ultrasonography

**Fig. 3:**
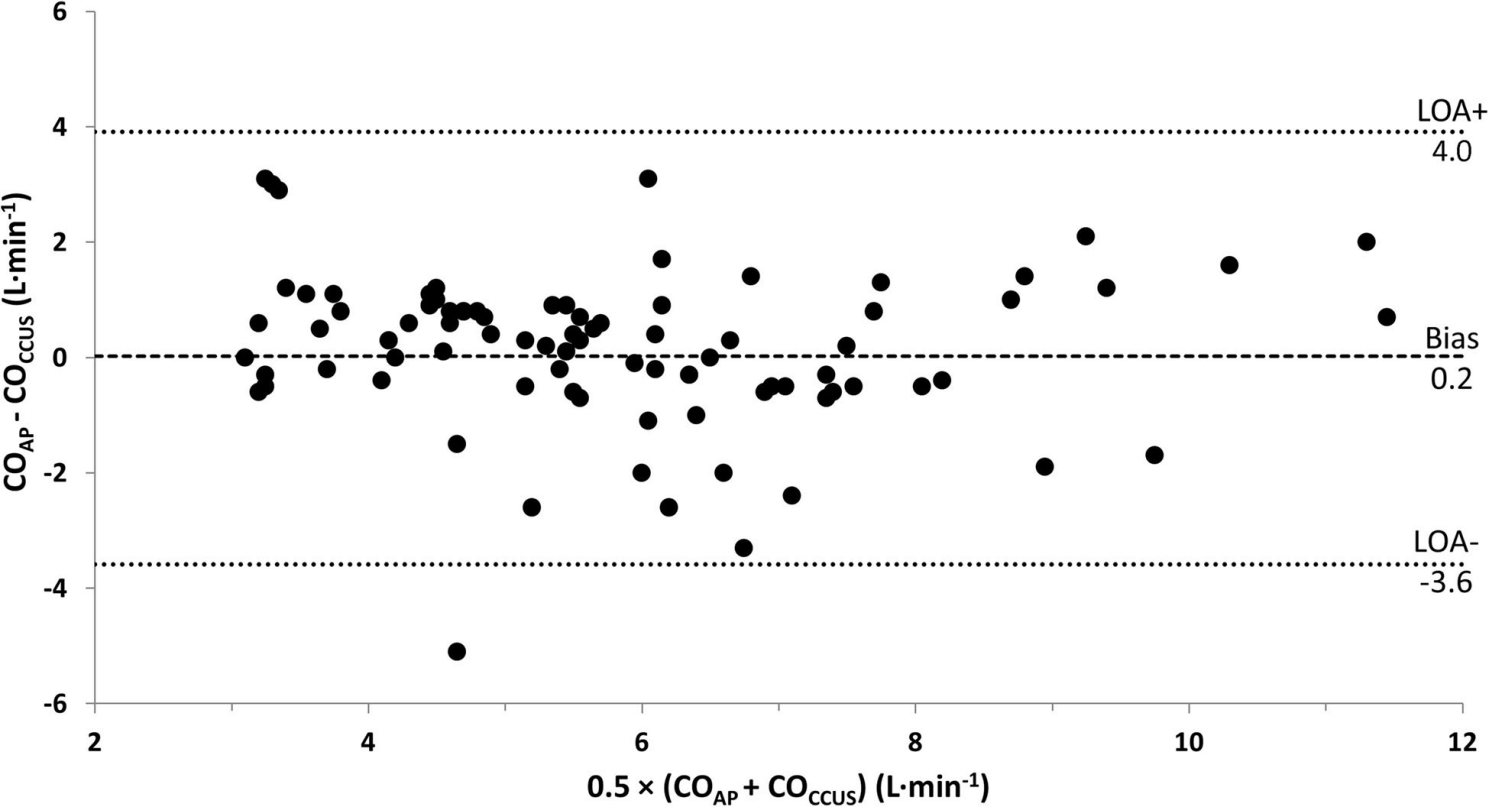
Bland-Altman plot for repeated measurements showing the comparison between CO_AP_ and CO_CCUS_. The mean bias between CO_AP_ and CO_CCUS_ and the upper and lower limits of agreement (LOA) are presented. Abbreviations: CO_AP_, cardiac output measured using fourth-generation FloTrac™ algorithm; CO_CCUS_, cardiac output measured by critical care ultrasonography

The characteristics of the 17 included patients are shown in Table 1 (and Additional file 2: Table S1). The mean CO_AP_ and CO_CCUS_ for 89 paired measurements were 5.9 ± 1.9 L min^−1^ and 5.7 ± 2.0 L min^−1^, respectively (*p* = 0.24). A significant correlation was observed for all CO measurements (*r*^2^ = 0.60, *p* < 0.001) (Fig. 2). Bias was 0.2 L min^−1^ (95% CI − 0.2 to 0.6), with LOA of − 3.6 L min^−1^ (95% CI − 4.3 to − 2.9) to 4.0 L min^−1^ (95% CI 3.3 to 4.7) (Fig. 3). Plotting a regression line in the Bland-Altman plot gave no arguments for proportional bias (line not shown). The overall percentage error was 65.6% (95% CI 53.2 to 77.3). Individual cardiac output measurements for each patient are provided in Additional file 3: Table S2.

**Table 1:** Patient characteristics

Patient characteristics (*n* = 17)		
Age (years)		65 (9)
Male gender, *n* (%)		14 (82%)
Body mass index (kg m^−2^)		25.7 (4.7)
Clinical characteristics on study inclusion		
Heart rate (bpm)		95 (26)
Systolic arterial pressure (mmHg)		102 (15)
Diastolic arterial pressure (mmHg)		55 (6)
Mean arterial pressure (mmHg)		69 (7)
Norepinephrine therapy, *n* (%)		16 (94%)
Norepinephrine dose (μg kg^−1^ min^−1^)		0.13 (0.05, 0.38)
Mechanical ventilation, *n* (%)		12 (71%)
Positive end-expiratory pressure (cm H_2_O)		8 (6, 9)
AVPU score, *n* (%)	Alert	2 (12%)
	Verbal	4 (24%)
	Passive	1 (6%)
	Unresponsive	10 (58%)
Mottling score, *n* (%)	None	3 (19%)
	Modest	2 (13%)
	Mild	7 (43%)
	Moderate	3 (19%)
	Severe	1 (6%)
Urine output (mL kg^−1^ h^−1^)		0.49 (0.26, 0.66)
Lactate (mmol L^−1^)		1.7 (1.4, 3.4)
APACHE IV—score (points)		92 (32)
SAPS II— (points)		56 (17)

Data are presented as the mean and standard deviation, median and interquartile ranges or as absolute frequencies with percentages as appropriate

*Abbreviations*: *AVPU* alert, verbal, pain, unresponsive; *APACHE* Acute Physiology And Chronic Health Evaluation; *SAPS* Simplified Acute Physiology Score

### Trending ability

**Fig. 4:**
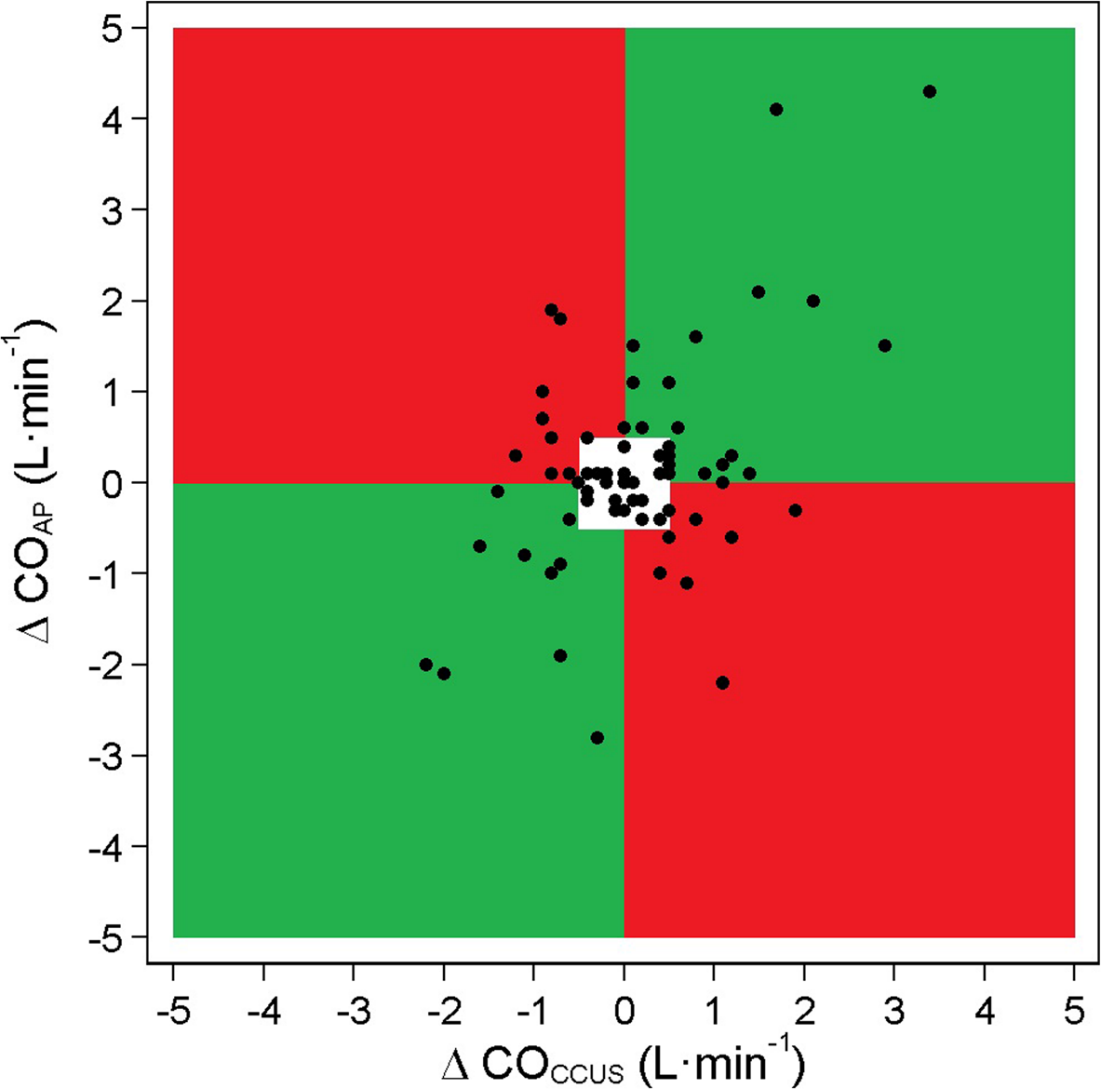
Four-quadrant plot showing the trend of CO_AP_ versus CO_CCUS_. Exclusion zone of 0.5 L min^−1^ (white area). Abbreviations: CO_AP_, cardiac output measured using fourth-generation FloTrac™ algorithm; CO_CCUS_, cardiac output measured by critical care ultrasonography

For assessment of trending ability 72 paired measurements were analysed. Trending of measurements was evaluated using a four-quadrant plot (Fig. 4). Forty-five paired measurements showed a clinically relevant change, which was defined as larger than 0.5 L min^−1^. The concordance rate was 64.4%.

## Discussion

In this prospective observational study, agreement and trending ability of CO_AP_ was compared with CO_CCUS_ in critically ill patients with circulatory shock. CO_AP_ showed a low bias of 0.2 L min^−1^ but a large percentage error of 65.6% when compared with CO_CCUS_, indicating disagreement [20]. Trending ability was poor with a concordance rate of 64.4%. The new FloTrac™ algorithm should not be used for diagnosis or guidance of treatment in critically ill patients with circulatory shock.

### Interpretation

There are no data on the reliability of CO measurements with the fourth-generation FloTrac™ software algorithm in critically ill patients with shock as of yet. The main concern with the previous version(s) of the APCO algorithm was the lack of reliability in tracking CO changes after hemodynamic interventions or in patients with sepsis [7, 22].

The low bias and the high percentage error of CO measurements are in accordance with results from another study, which tested the fourth-generation algorithm for tracking CO measurements after administration of phenylephrine to increase vasomotor tone in patients prior to cardiac surgery (bias − 0.7 L min^−1^; percentage error 55.4%) [23]. Concordance rate for trending ability was 87% which was higher than in our study. In that study, the chosen reference technique for measuring CO was thermodilution.

In a more recent study in patients undergoing cardiac surgery, the new FloTrac™ algorithm also showed lack of agreement and trending ability (bias − 0.4 L min^−1^; percentage error 37.1%; concordance rate 76%) [24]. The reference technique was thermodilution and in that study, bias was influenced by systemic vascular resistance.

Another study tested the fourth-generation FloTrac™ algorithm in patients undergoing abdominal aortic aneurysm surgery and also found a low bias and high percentage error (bias 0.4 L min^−1^; percentage error 46.7%) of CO measurements [25]. The concordance rate for trending ability was 26.9% before and after aortic clamping and 47.3% before and after first unclamping of the iliac artery. The reference technique chosen in this study was transoesophageal echocardiography.

Advanced hemodynamic monitoring techniques are currently used to identify the type of shock, to guide choices of interventions and to evaluate the response to therapy. Less invasive hemodynamic monitoring techniques such as APCO are currently not recommended for use in patients with shock, especially when receiving vasopressors [2, 26]. Our findings support this statement.

### Implications and generalizability

Even though CO monitoring is considered a cornerstone in diagnosing and managing circulatory shock, the sequential evaluation of the hemodynamic state during shock is only a level 1 recommendation based on low quality of evidence [2].

The abovementioned studies validating the new fourth-generation FloTrac™ algorithm were performed in different target populations and contained different reference techniques, which limit comparability. There is a concern about the interchangeability of CO_CCUS_ and CO measurements by thermodilution, and tracking ability of the two methods has only been scarcely assessed and needs evaluation by larger studies [27].

### Considerations and limitations

There are several considerations and limitations when interpreting the results of our study. First, since only parallel and no serial CO measurements were performed for each time point, the precision of individual measurements could not be assessed. While only few studies determined the precision of the CCUS and FloTrac™ technologies, it is a given that both methods have some degree of variation which influences precision of agreement [28]. This might influence—and possibly overestimate—the observed bias and precision to an unknown extent, since the precision of the CCUS as reference method was not incorporated.

Second, a stepwise approach and checklist for the complete presentation of CO method comparison research have been published [10]. This checklist includes a design study phase where it is encouraged that criteria for acceptable bias and LOA or percentage error are defined, and a sample size calculation should be performed prior to the conduct of method comparison studies. In our study, we defined clinically acceptable limits based on available literature, but we did not specify a sample size in advance. The current study could serve as a pilot for a further validation study.

Third, during the study period, we included only 17 patients. Patients with circulatory shock were eligible only if they were expected to stay for longer than 48 h and if it was possible to perform CCUS. We chose this definition to ensure that a complete picture of shock treatment could be presented which allowed for the best comparison between the two methods. Last, CCUS was used as a reference technique in our study despite pulmonary or transpulmonary thermodilution being the gold standard for CO method comparison studies [10]. Therefore, we cannot prove direct superiority of either method. In order to do this, a comparison with a thermodilution method will have to be performed. We chose CCUS as reference because it is currently the first-line evaluation modality in patients with circulatory shock and also because it is widely available and used in the ICU for diagnostic purposes [2, 29]. However, images required to make CO_CCUS_ measurements are unobtainable in up to 20% of patients [30].

FloTrac™ measurements of CO are still not recommended in critically ill patients [5, 6], and further clinical studies comparing minimally invasive techniques for CO estimation with a reference technique are needed for further validation of these techniques and also for extending applicability to other types of patients, who were initially not the target population.

## Conclusions

In critically ill patients with circulatory shock, there was disagreement and clinically unacceptable trending ability between values of cardiac output obtained by uncalibrated arterial pressure waveform analysis and critical care ultrasonography.

## Additional files

Additional file 1: STROBE Statement-Checklist of items that should be included in reports of cohort studies. Checklist of items that should be included in reports of cohort studies according to the STROBE statement. (DOCX 42 kb)

Additional file 2: **Table S1.** Detailed patient characteristics. Table showing extended patient characteristics to further describe the study population. (DOCX 15 kb)

Additional file 3: **Table S2.** Cardiac output measurements with critical care ultrasonography and FloTrac. Overview of each cardiac output measurement performed with critical care ultrasonography and FloTrac. (DOCX 24 kb)

## Abbreviations

APACHE: Acute Physiology And Chronic Health Evaluation
APCO: Arterial Pressure waveform analysis method to estimate Cardiac Output
CCUS: Critical care ultrasonography
CO: Cardiac output
CO_AP_: Cardiac output measured using fourth-generation FloTrac™ algorithm
CO_CCUS_: Cardiac output measured using critical care ultrasonography
ICU: Intensive care unit
LOA: Limits of agreement
MAP: Mean arterial pressure
PAC: Pulmonary artery catheter
SAPS: Simple Acute Physiology Score
SICS: Simple Intensive Care Studies
STROBE: STrengthening the Reporting of OBservational studies in Epidemiology
SV: Stroke volume
