# Supplementary material for: Disagreement in cardiac output measurements between fourth-generation FloTrac and critical care ultrasonography in patients with circulatory shock: a prospective observational study
Source: J Intensive Care. 2019 Apr 11;7:21. doi: 10.1186/s40560-019-0373-5 (PMC6460822; doi:10.1186/s40560-019-0373-5)
Supplement: Supplementary file 2 — Table S1. Detailed patient characteristics. Table showing extended patient characteristics to further describe the study population. (DOCX 15 kb) [file 40560_2019_373_MOESM2_ESM.docx]

Table S1. Detailed patient characteristics (*n*=17)

| Study ID | Gender | Age | Diagnosis at Admission | Type of Shock | APACHE IV score | SAPS-II | 90-day mortality |
| --- | --- | --- | --- | --- | --- | --- | --- |
| 1 | Male | 68 | Sepsis | Distributive | 111 | 68 | No |
| 2 | Male | 61 | Shock e.c.i. | Distributive & Obstructive | 176 | 104 | Yes |
| 3 | Male | 48 | Haemodynamic instability after liver biopsy | Hypovolemic | 102 | 60 | Yes |
| 4 | Male | 70 | Left ventricular free wall rupture | Cardiogenic | 68 | 39 | No |
| 5 | Male | 82 | Myocardial infarction | Cardiogenic | 46 | 50 | Yes |
| 6 | Female | 78 | Sepsis | Distributive | 107 | 55 | No |
| 7 | Male | 50 | Sepsis | Distributive | 122 | 66 | Yes |
| 8 | Female | 54 | Sepsis | Distributive | 114 | 71 | No |
| 9 | Female | 70 | HIPEC | Distributive | 57 | 55 | No |
| 10 | Male | 75 | Intestinal perforation after mechanical ileus | Distributive | 87 | 58 | Yes |
| 11 | Male | 63 | Sepsis | Distributive | Missing | 29 | No |
| 12 | Male | 57 | OHCA | Cardiogenic | 83 | 52 | No |
| 13 | Male | 59 | Sepsis | Distributive | 60 | 45 | No |
| 14 | Male | 69 | Sepsis | Distributive | 100 | 63 | No |
| 15 | Male | 65 | Myocardial infarction | Cardiogenic | 86 | 59 | No |
| 16 | Male | 67 | Sepsis | Distributive | 69 | 36 | No |
| 17 | Male | 66 | Sepsis | Distributive | 78 | 50 | Yes |

Abbreviations: APACHE = Acute Physiology And Chronic Health Evaluation; SAPS = Simple Acute Physiology Score; HIPEC = Hyperthermic Intraperitoneal Chemotherapy; OHCA = Out of Hospital Cardiac Arrest.
