# Supplementary material for: Disagreement in cardiac output measurements between fourth-generation FloTrac and critical care ultrasonography in patients with circulatory shock: a prospective observational study
Source: J Intensive Care. 2019 Apr 11;7:21. doi: 10.1186/s40560-019-0373-5 (PMC6460822; doi:10.1186/s40560-019-0373-5)
Supplement: Supplementary file 3 — Table S2. Cardiac output measurements with critical care ultrasonography and FloTrac. Overview of each cardiac output measurement performed with critical care ultrasonography and FloTrac. (DOCX 24 kb) [file 40560_2019_373_MOESM3_ESM.docx]

Table S2. Cardiac output measurements with critical care ultrasonography and FloTrac

| **Measurement number** | **Cardiac output measured by CCUS** (**L min^-1^)** | **Cardiac output measured by FloTrac** (**L min^-1^)** | **Difference between measurements** | **Mean of both measurements** |
| --- | --- | --- | --- | --- |
| **Study ID 1. 68 year old male with distributive shock** | | | | |
| 1 | 5.2 | 5.9 | 0.7 | 5.6 |
| 2 | 5.3 | 7.0 | 1.7 | 6.2 |
| 3 | 6.0 | 5.9 | -0.1 | 6.0 |
| 4 | 5.4 | 5.5 | 0.1 | 5.5 |
| 5 | 5.4 | 5.9 | 0.5 | 5.7 |
| 6 | 5.9 | 6.3 | 0.4 | 6.1 |
| **Study ID 2. 61 year old male with distributive and obstructive shock** | | | | |
| 1 | 5.0 | 5.3 | 0.3 | 5.2 |
| 2 | 5.4 | 4.9 | -0.5 | 5.2 |
| 3 | 5.4 | 5.5 | 0.1 | 5.5 |
| 4 | 5.9 | 5.2 | -0.7 | 5.6 |
| 5 | 5.0 | 5.9 | 0.9 | 5.5 |
| 6 | 5.5 | 5.3 | -0.2 | 5.4 |
| **Study ID 3. 48 year old male with hypovolemic shock** | | | | |
| 1 | 6.9 | 5.9 | -1.0 | 6.4 |
| 2 | 8.4 | 8.0 | -0.4 | 8.2 |
| 3 | 6.2 | 6.0 | -0.2 | 6.1 |
| 4 | 5.3 | 7.0 | 1.7 | 6.2 |
| 5 | 7.2 | 6.7 | -0.5 | 7.0 |
| **Study ID 4. 70 year old male with cardiogenic shock** | | | | |
| 1 | 4.3 | 5.1 | 0.8 | 4.7 |
| 2 | 4.3 | 5.1 | 0.8 | 4.7 |
| 3 | 3.9 | 5.0 | 1.1 | 4.5 |
| 4 | 3.9 | 5.1 | 1.2 | 4.5 |
| 5 | 4.0 | 4.9 | 0.9 | 4.5 |
| 6 | 4.4 | 5.2 | 0.8 | 4.8 |
| 7 | 4.3 | 4.9 | 0.6 | 4.6 |
| 8 | 4.0 | 5.0 | 1.0 | 4.5 |
| 9 | 4.5 | 5.2 | 0.7 | 4.9 |
| **StudyID 5. 82 year old male with cardiogenic shock** | | | | |
| 1 | 4.5 | 4.6 | 0.1 | 4.6 |
| 2 | 4.0 | 4.6 | 0.6 | 4.3 |
| 3 | 4.0 | 4.3 | 0.3 | 4.2 |
| 4 | 4.0 | 4.3 | 0.3 | 4.2 |
| **StudyID 6. 78 year old female with distributive shock** | | | | |
| 1 | 8.3 | 5.9 | -2.4 | 7.1 |
| 2 | 7.5 | 4.9 | -2.6 | 6.2 |
| 3 | 7.2 | 2.1 | -5.1 | 4.7 |
| 4 | 6.5 | 3.9 | -2.6 | 5.2 |
| 5 | 7.0 | 5.0 | -2.0 | 6.0 |
| 6 | 8.4 | 5.1 | -3.3 | 6.8 |
| 7 | 7.6 | 5.6 | -2.0 | 6.6 |
|  |  |  |  |  |
| **StudyID 7. 50 year old male with distributive shock** | | | | |
| 1 | 11.1 | 11.8 | 0.7 | 11.5 |
| 2 | 9.5 | 11.1 | 1.6 | 10.3 |
| 3 | 10.6 | 8.9 | -1.7 | 9.8 |
| 4 | 9.9 | 8.0 | -1.9 | 9.0 |
| **StudyID 8. 54 year old female with distributive shock** | | | | |
| 1 | 8.1 | 9.5 | 1.4 | 8.8 |
| 2 | 7.4 | 7.6 | 0.2 | 7.5 |
| 3 | 8.2 | 9.2 | 1.0 | 8.7 |
| 4 | 7.1 | 8.4 | 1.3 | 7.8 |
| 5 | 8.3 | 7.8 | -0.5 | 8.1 |
| **StudyID 9. 70 year old female with distributive shock** | | | | |
| 1 | 3.0 | 4.1 | 1.1 | 3.6 |
| 2 | 3.4 | 4.2 | 0.8 | 3.8 |
| 3 | 3.2 | 4.3 | 1.1 | 3.8 |
| 4 | 3.4 | 3.9 | 0.5 | 3.7 |
| 5 | 2.8 | 4.0 | 1.2 | 3.4 |
| **Study ID 10. 75 year old male with distributive shock** | | | | |
| 1 | 2.9 | 3.5 | 0.6 | 3.2 |
| 2 | 3.8 | 3.6 | -0.2 | 3.7 |
| 3 | 4.3 | 3.9 | -0.4 | 4.1 |
| 4 | 5.4 | 3.9 | -1.5 | 4.7 |
| 5 | 4.2 | 4.2 | 0.0 | 4.2 |
| **StudyID 11. 63 year old male with distributive shock** | | | | |
| 1 | 4.9 | 5.8 | 0.9 | 5.4 |
| 2 | 7.8 | 7.3 | -0.5 | 7.6 |
| 3 | 5.8 | 5.2 | -0.6 | 5.5 |
| 4 | 5.4 | 5.7 | 0.3 | 5.6 |
| 5 | 8.8 | 10.0 | 1.2 | 9.4 |
| **StudyID 12. 57 year old male with cardiogenic shock** | | | | |
| 1 | 3.4 | 3.1 | -0.3 | 3.3 |
| 2 | 3.5 | 2.9 | -0.6 | 3.2 |
| 3 | 3.5 | 3.0 | -0.5 | 3.3 |
| 4 | 3.1 | 3.1 | 0.0 | 3.1 |
| **StudyID 13. 59 year old male with distributive shock** | | | | |
| 1 | 4.2 | 5.0 | 0.8 | 4.6 |
| 2 | 4.7 | 5.1 | 0.4 | 4.9 |
| 3 | 5.3 | 5.7 | 0.4 | 5.5 |
| 4 | 4.5 | 7.6 | 3.1 | 6.1 |
| **StudyID 14. 69 year old male with distributive shock** | | | | |
| 1 | 6.6 | 5.5 | -1.1 | 6.1 |
| 2 | 5.2 | 5.4 | 0.2 | 5.3 |
| 3 | 5.4 | 6.0 | 0.6 | 5.7 |
| 4 | 6.5 | 6.2 | -0.3 | 6.4 |
| 5 | 8.2 | 10.3 | 2.1 | 9.3 |
| 6 | 10.3 | 12.3 | 2.0 | 11.3 |
|  |  |  |  |  |
| **StudyID 15. 65 year old male with cardiogenic shock** | | | | |
| 1 | 1.9 | 4.8 | 2.9 | 3.4 |
| 2 | 1.7 | 4.8 | 3.1 | 3.3 |
| 3 | 1.8 | 4.8 | 3.0 | 3.3 |
| **StudyID 16. 67 year old male with distributive shock** | | | | |
| 1 | 7.5 | 7.2 | -0.3 | 7.4 |
| 2 | 7.7 | 7 | -0.7 | 7.4 |
| 3 | 7.3 | 6.8 | -0.5 | 7.1 |
| 4 | 7.2 | 6.6 | -0.6 | 6.9 |
| 5 | 7.3 | 8.1 | 0.8 | 7.7 |
| **StudyID 17. 66 year old male with distributive shock** | | | | |
| 1 | 6.1 | 7.5 | 1.4 | 6.8 |
| 2 | 6.5 | 6.5 | 0.0 | 6.5 |
| 3 | 5.7 | 6.6 | 0.9 | 6.2 |
| 4 | 6.5 | 6.2 | -0.3 | 6.4 |
| 5 | 6.5 | 6.8 | 0.3 | 6.7 |
| 6 | 7.7 | 7.1 | -0.6 | 7.4 |

Abbreviations: CCUS; critical care ultrasonography
